# Interobserver variation in the classification of tumor deposits in rectal cancer—is the use of histopathological characteristics the way to go?

**DOI:** 10.1007/s00428-021-03197-0

**Published:** 2021-09-04

**Authors:** Nelleke P. M. Brouwer, A. C. Lord, M. Terlizzo, A. C. Bateman, N. P. West, R. Goldin, A. Martinez, N. A. C. S. Wong, M. Novelli, I. D. Nagtegaal, G. Brown

**Affiliations:** 1 Department of Pathology, Radboud University Medical Centre, Geert-Grooteplein Zuid 10, 6500 HB Nijmegen, The Netherlands; 2 Department of Gastrointestinal Imaging, Royal Marsden NHS Foundation Trust, London, UK; 3 Department of Pathology, University Hospital Southampton, Southampton, UK; 4 Department of Pathology, University of Leeds, Leeds, UK; 5 Department of Pathology, Imperial College, London, UK; 6 Department of Pathology, St Marks Hospital, London, UK; 7 Department of Pathology, University of Bristol, Bristol, UK; 8 Department of Pathology, University College London, London, UK

**Keywords:** Rectal cancer, Tumor deposit, Lymph node metastasis, Histopathology, Interobserver variability, TNM staging

## Abstract

The focus on lymph node metastases (LNM) as the most important prognostic marker in colorectal cancer (CRC) has been challenged by the finding that other types of locoregional spread, including tumor deposits (TDs), extramural venous invasion (EMVI), and perineural invasion (PNI), also have significant impact. However, there are concerns about interobserver variation when differentiating between these features. Therefore, this study analyzed interobserver agreement between pathologists when assessing routine tumor nodules based on TNM 8. Electronic slides of 50 tumor nodules that were not treated with neoadjuvant therapy were reviewed by 8 gastrointestinal pathologists. They were asked to classify each nodule as TD, LNM, EMVI, or PNI, and to list which histological discriminatory features were present. There was overall agreement of 73.5% (κ 0.38, 95%-CI 0.33–0.43) if a nodal versus non-nodal classification was used, and 52.2% (κ 0.27, 95%-CI 0.23–0.31) if EMVI and PNI were classified separately. The interobserver agreement varied significantly between discriminatory features from κ 0.64 (95%-CI 0.58–0.70) for roundness to κ 0.26 (95%-CI 0.12–0.41) for a lone arteriole sign, and the presence of discriminatory features did not always correlate with the final classification. Since extranodal pathways of spread are prognostically relevant*,* classification of tumor nodules is important. There is currently no evidence for the prognostic relevance of the origin of TD, and although some histopathological characteristics showed good interobserver agreement, these are often non-specific. To optimize interobserver agreement, we recommend a binary classification of nodal versus extranodal tumor nodules which is based on prognostic evidence and yields good overall agreement.

**Supplementary Information:**

The online version contains supplementary material available at 10.1007/s00428-021-03197-0.

## Introduction

In the 1930s, Dukes described lymph node metastases (LNM) as a poor prognostic factor in rectal cancer [1], which led to the development of the prevailing hypothesis that metastatic disease is a direct result of lymphatic spread. However, recent evidence challenges the focus on LNM as the gateway to distant metastasis by showing that 65% of distant metastases were not associated with identifiable LNM [2]. Also, meta-analyses have confirmed and reinforced the significant prognostic value of other mechanisms of locoregional spread, with TD having the strongest impact [3–6].

Due to their prognostic importance, tumor deposits (TDs) have been incorporated into TNM staging since the 5^th^ edition, but their definition has changed over the years leading to confusion. TD may or may not be considered LNM and have been stratified according to size, contour, or the presence of histological structures [7–10]. With changing definitions of TD throughout different TNM editions, interobserver variability has also changed. Low interobserver variation (κ 0.84) was noted when identifying all nodules larger than 3 mm as TD (TNM 5) [11], whereas the definition based on contour (TNM6) led to high interobserver variation (κ 0.21) [11, 12]. The complex definition of TD in TNM7, based on the presence of histological structures, led to moderate interobserver variation (κ 0.48) when assessing challenging nodules [13].

The definition of TD has increased in complexity in TNM8 in an attempt to classify TD further based on their origin as LNM, extramural venous invasion (EMVI), and perineural invasion (PNI). TD should only be used in the absence of an identifiable origin. However, although LNM, EMVI, and PNI in themselves have prognostic impact, it is not clear whether subclassification of TD according to origin is relevant. Moreover, TNM 8 does not include guidelines about how to differentiate between TD and LNM, EMVI, or PNI, especially when multiple potential mechanisms of spread are noted within the same nodule [10]. Lastly, the interobserver variation during the assessment of these features has not been investigated in daily practice. Therefore, we aimed to assess interobserver variation among expert gastrointestinal pathologists regarding classification of locoregional spread in rectal cancer using TNM8 and cases taken from routine practice. In addition, we assessed which histological features were applied to discriminate between different types of locoregional spread.

## Methods

### Case selection

Hematoxylin and eosin (H&E) slides from 79 tumor nodules were selected from a retrospective cohort study and the MERCURY trial. The selection included 50 tumor nodules from patients that did not receive neoadjuvant treatment, and 29 nodules from patients that did. The tumor nodules had been reported as either LNMs or TDs in the original pathology report, and were therefore defined as discontinuous from the primary tumor. The patients were treated for rectal cancer between 2002 and 2003 in the Royal Marsden Hospital or Leeds General Infirmary (MERCURY trial), and 2011–2015 in the Royal Marsden Hospital (retrospective cohort) [14]. The nodules were selected by a member of the research team who was not a pathologist and made no attempt to select “challenging” cases. The H&E slides from the MERCURY trial were scanned using an Aperio Scanscope XT scanner (Leica Biosystems, Nussloch, Germany) at 20 × magnification and slides from the retrospective cohort were scanned using a Nanozoomer 2.0‐HT (Hamamatsu Corporation, Hamamatsu City, Japan) microscopic‐resolution scanner at 40 × magnification. The virtual slides were sent to 8 gastrointestinal pathologists and each pathologist was asked to categorize the nodules into TD, LNM, EMVI, or PNI (TNM8 definition). Pathologists were blinded to the original report, other participants’ responses, and patient outcomes and could only examine a single H&E-stained slide without the possibility of using additional stains or techniques.

A list of histological features which could be seen as potential discriminatory factors was collated, including the following histological features: round shape, capsule, peripheral lymphocyte ring, lymphoid follicles, subcapsular sinus, vessel wall, “lone arteriole” sign, vessel encasement, and perineural invasion. Pathologists were asked to record which of these features were present in each nodule examined before recording their final classification.

### Statistical analysis

In general, only the nodules that did not receive neoadjuvant treatment were included in the analyses. The nodules that were treated with neoadjuvant therapy were analyzed only for the Fleiss multi-rater kappa. The Fleiss multi-rater kappa with a 95% confidence interval was used for the evaluation of interobserver variation and calculated in R Studio (version 3.6.2). The agreement was classified as poor (< 0.0), slight (0.00–0.20), fair (0.21–0.40), moderate (0.41–60), substantial (0.61–0.80), and almost perfect (0.81–1.00) [15]. Sunburst charts were constructed to provide a hierarchal overview of the presence of different discriminatory features. This was done for lymphatic features (capsule, peripheral lymphocyte ring, lymphoid follicles, subcapsular sinus) and venous features (vessel wall, lone arteriole sign, encasing vessel). Also, the number of lymphatic, venous, or perineural features was plotted in sunburst chart for nodules that were scored as TD. Analyses were performed using Microsoft Excel (2016).

## Results

For this study, 79 tumor nodules from 41 patients were reviewed by 8 gastrointestinal pathologists, from 8 different institutions. The pathologists had a median of 18 years’ (range 12–38) experience in specialist practice. From all cases, 50 were not treated with neoadjuvant therapy (radiotherapy or chemoradiotherapy). For all analyses, only the cases without neoadjuvant treatment were used, unless stated otherwise.

### Interobserver agreement

**Fig. 1:**
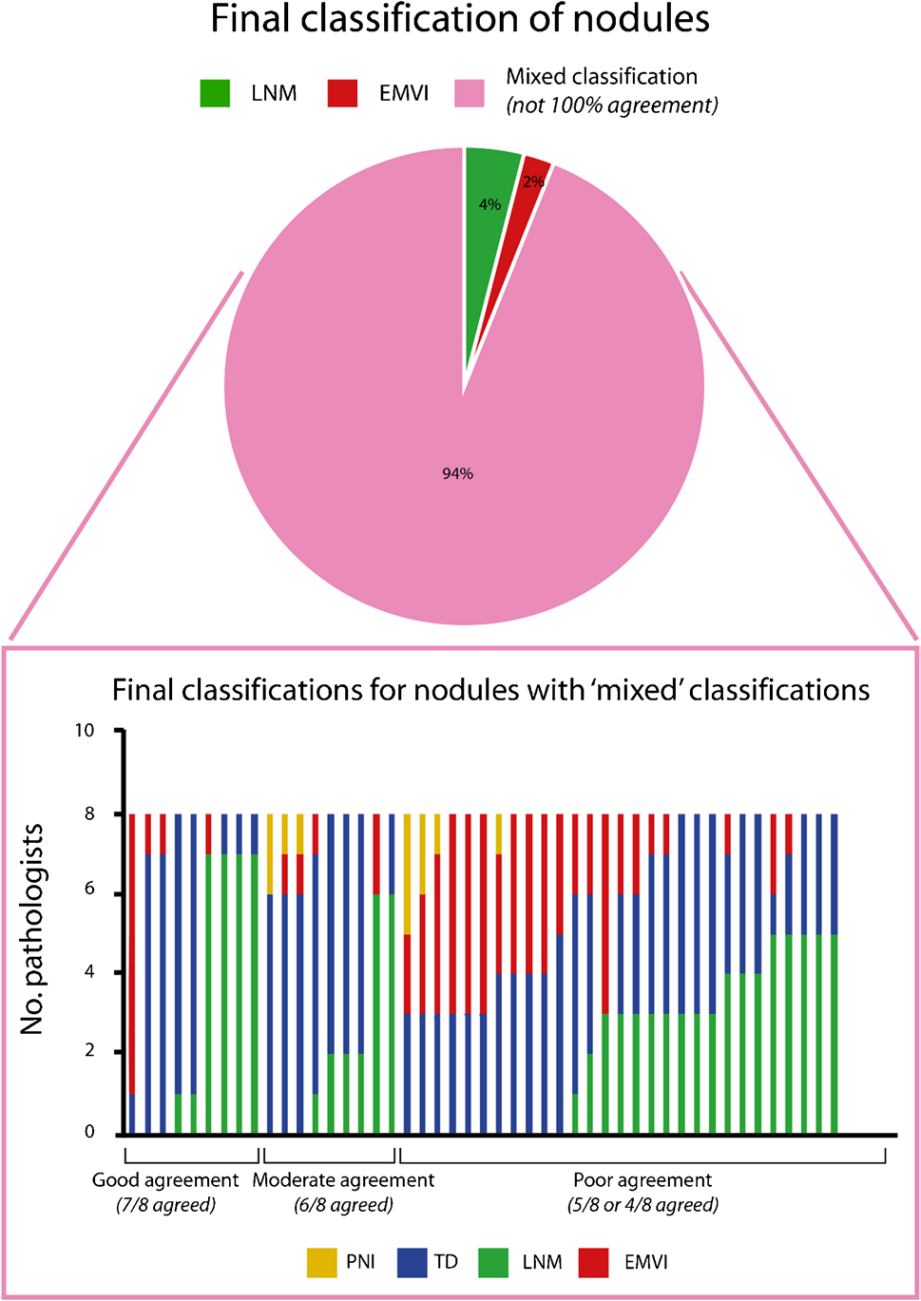
The final classification of the nodules. The percentage of nodules with 100% agreement on the classification as LNM, TD, EMVI, or PNI and cases with “mixed” classifications from which a more in-depth overview is shown in the bar chart. Abbreviations: LNM, lymph node metastasis; TD, tumor deposit; EMVI, extramural venous invasion; PNI, perineural invasion

When tumor nodules were given a binary classification of “nodal” or “non-nodal” origin, the overall agreement was 73.5% with a κ of 0.38 (95%-CI 0.33–0.43), indicating moderate agreement. More detailed classification of nodules as LNM, TD, PNI, or EMVI yielded a κ of 0.27 (95%-CI 0.23–0.31) and an overall agreement of 52.2%. For nodules from patients treated with neoadjuvant therapy, the mean percentage agreement also improved when a binary classification was used (supplementary table 1). When looking at the final classification of nodules, 6% (3/50) were scored with complete agreement among all 8 pathologists while 94% had varying classifications. Of these cases, 9 nodules had good agreement (7/8), 9 had moderate agreement (6/8), and 29 had poor agreement (5/8 or 4/8) (Fig. 1).

The overall agreement and κ for the discriminatory features are shown in Table 1. The agreement varied significantly between the different histological features, with substantial agreement for the roundness of a nodule (κ 0.64) whereas the presence of a subcapsular sinus showed only slight agreement (κ 0.20).

**Table 1:** Interobserver agreement for discriminatory features

			Kappa agreement	
Histological feature	Times feature was scored as present (of 400 scores)*	Mean percentage agreement	κ	95% confidence interval
Round shape	232	82.4%	0.64	(0.58–0.70)
Perineural invasion	55	87.4%	0.47	(0.25–0.68)
Peripheral ring	140	73.4%	0.42	(0.34–0.50)
Lymphoid follicles	77	80.1%	0.36	(0.20–0.52)
Capsule	130	71.1%	0.34	(0.25–0.43)
Vessel wall	89	76.6%	0.32	(0.18–0.47)
Lone arteriole sign	87	72.0%	0.26	(0.12–0.41)
Encasing vessel	54	81.7%	0.22	(0.00–0.43)
Subcapsular sinus	14	95.5%	0.20	(− 0.30–0.70)

Agreement among pathologists for the presence or absence of discriminatory features. All 8 pathologists scored the different histological features for all 50 nodules, yielding a total of 400 scores per histological feature

^*^It is important to note that kappa may not be reliable for rare observations, such as the presence of a subcapsular sinus (14/400). Kappa is affected by the prevalence of the finding under consideration, much like predictive values are affected by the prevalence of the disease under considerations. Therefore, for rare findings, very low values of kappa may not necessarily reflect low rates of overall agreement^21^.

### Sunburst charts

**Fig. 2:**
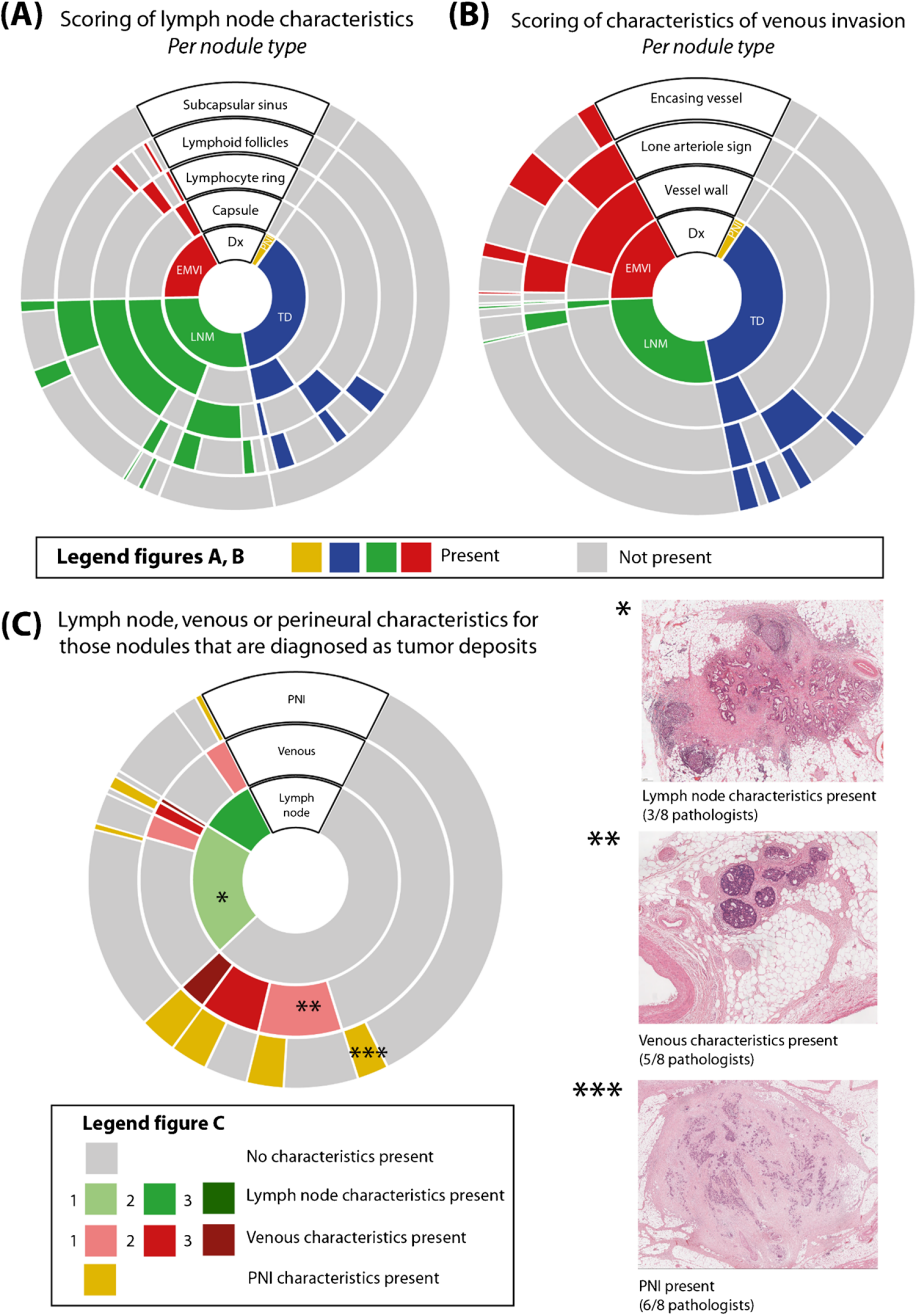
Sunburst charts visualizing the different histological characteristics scored by the pathologists in a hierarchical manner. The final classification of the nodules is shown in the inner circle and the presence (color) or absence (gray) of different histological structures is shown in the other layers of the chart. Reading the charts from the middle outwards, the final classification (PNI, TD, LNM, EMVI) as well as the presence of histological features can be seen for every individual nodule. **A** Lymph node features; capsule, lymphocyte ring, lymphoid follicles, subcapsular sinus. **B** Features of venous invasion; vessel wall, lone arteriole sign, encasing vessel. **C** The number of features of different origins (lymph node, vein, nerve) for nodules classified as TD. Examples of a specific category are indicated with asterisks. (A) Scoring of lymph node characteristics *Per nodule type* . (B) Scoring of characteristics of venous invasion *Per nodule type*. (C) Lymph node, venous or perineural characteristics for those nodules that are diagnosed as tumor deposits. Abbreviations: LNM, lymph node metastasis; TD, tumor deposit; EMVI, extramural venous invasion; PNI, perineural invasion

To visualize the different histological features that the pathologist scored, sunburst charts were constructed for lymphatic and venous features (Fig. 2A, B). The inner circle shows the proportions of the final classification (EMVI, PNI, TD, LNM), with deeper hierarchy levels in the surrounding circles. For example, in Fig. 2A, the final classification of a LNM was correlated with the presence (in green) or absence (in gray) of the different histological features. First, the presence of a capsule was plotted, stratifying the group of LNM into two groups. Then, for both of these groups, the presence or absence of a lymphocyte ring was plotted, and so on. This layering method enables the detailed visualization of all different sets of histological characteristics that were scored within the LNM cases. The presence of lymphatic features was most often scored for those nodules with the classification LNM, as can be expected (Fig. 2A). However, pathologists scored lymphatic features as being present in all other types of final classification as well, with the largest proportion in the TD group.

The sunburst chart including features of venous invasion for the different final classifications shows that these features were most often scored as present in the nodules that were called EMVI. Similarly to lymph node features, features of venous invasion were also scored in nodules with the other final classifications, with the largest proportion in nodules that were called TD (Fig. 2B).

To see why pathologists call certain tumor nodules TD, a sunburst chart was constructed for cases classified as TD with the different features for LNM, EMVI, and PNI in the different rings. Approximately 1/3 of the cases showed no features of any of the groups (all three levels are gray in the chart), 1/3 showed features from multiple origins, and, interestingly, 1/3 were called a TD despite the fact that only features from one specific origin were scored. Examples of this last group are shown with asterisks to the right of the chart (Fig. 2C).

### Illustration of cases

**Fig. 3:**
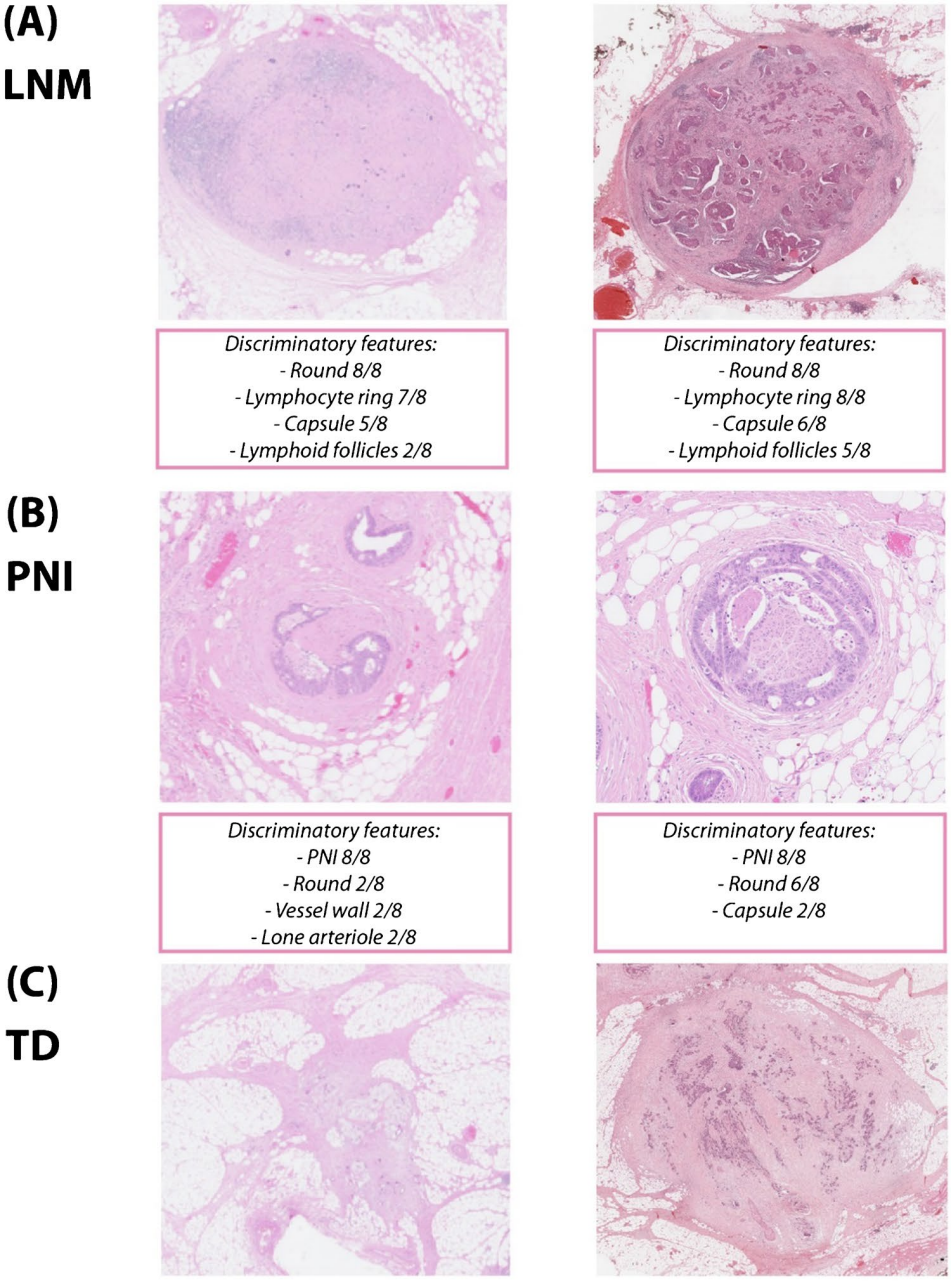
Examples of nodules with 100% agreement on the final classification, including the discriminatory features for cases classified as PNI or LNM. **A** Final classification LNM. **B** Final classification PNI. **C** Final classification TD. Abbreviations: LNM, lymph node metastasis; TD, tumor deposit; PNI, perineural invasion

**Fig. 4:**
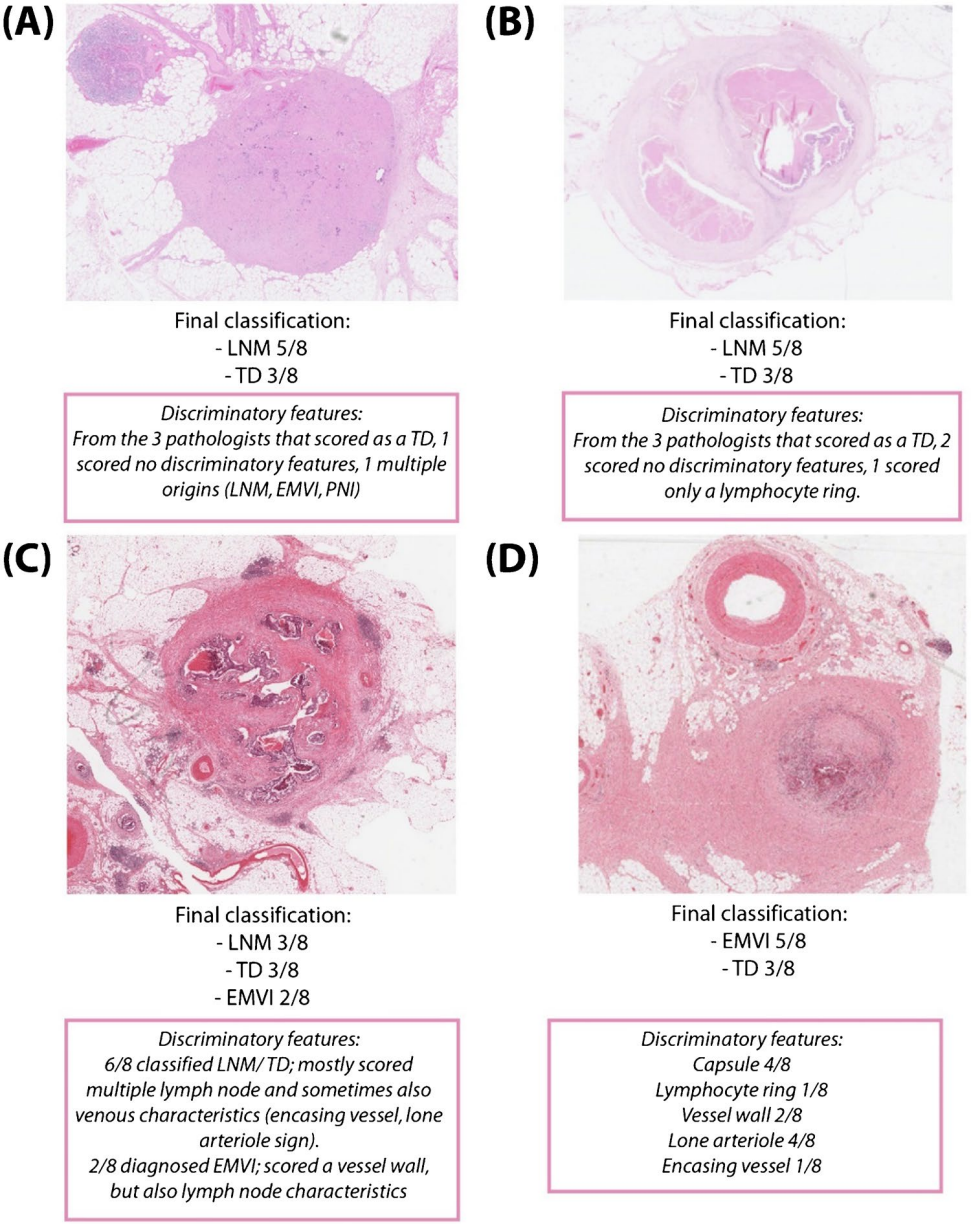
Examples of cases with incomplete agreement**.** The final classifications as well as a description of the discriminatory features are shown. **A** Final classifications LNM and TD. **B** Final classifications LNM and TD. **C** Final classifications LNM, TD, and EMVI. **D** Final classifications EMVI and TD. Abbreviations: LNM, lymph node metastasis; TD, tumor deposit; EMVI, extramural venous invasion; PNI, perineural invasion

To give more insight into the type of nodules that were scored, 10 cases were selected of which 6 had 100% agreement among pathologists regarding the final classification and 4 had incomplete agreement (Figs. 3 and 4).

## Discussion

This study highlights the difficulties in obtaining good interobserver agreement in distinguishing different types of locoregional spread using the TNM8 definition in daily practice. Reliable distinction between nodal and extranodal deposits yielded fairly good overall agreement of 73.5% (κ 0.38) which decreased to 52.2% (κ 0.27) when TDs were subdivided into LNM, TD, EMVI or PNI. The results show that suspicious, extranodal, tumor nodules can be identified with fairly good consensus when a binary classification of nodal versus extranodal tumor nodules is applied.

The results from this study are similar to previous findings when assessing “challenging” deposits using TNM 7 [13]. In this study, where nodules were classified as LNM or TD, 44% of cases showed “complete agreement” among pathologists in their final classification (κ 0.48). In comparison, in our study, there was only complete agreement in 6% of the cases when deposits were categorized as LNM, TD, EMVI, or PNI (κ 0.27), which increased to 40% (κ 0.38) when nodal versus non-nodal classification was used. This shows that interobserver agreement does not improve using TNM 8 definitions or “easier” nodules from daily practice.

The interobserver variability found in this study can be explained by the complex and subjective definition of tumor nodules in TNM8. Since previous definitions were based on weak and unsubstantial data [16], TNM7 incorporated TD into a new N category (N1c), defining them as “any cancerous nodule, located in the lymph drainage area of the peritumoral fatty tissue, irrespective of size or shape, as long as there is histologically proven absence of residual lymphatic tissue” [9]. The definition has increased in complexity in the TNM8 in an attempt to refine the classification, adding that “histological evidence of residual lymph node or identifiable vascular or neural structures should be absent” and that “if a vessel wall is identifiable on H&E, elastic or other stains, it should be classified as venous invasion (V1/2) or lymphatic invasion (L1). Similarly, if neural structures are identifiable, the lesion should be classified as perineural invasion (Pn1)” [10]. Some pathologists involved in this study would only apply this caveat to nodules which are entirely confined to a vein or to the perineurium, and not to nodules containing some evidence of EMVI or PNI but with the majority of tumor cells lying within the fat. Others said they took the statement at face value and would not classify any nodule with evidence of underlying origin as a TD.

The definition of TD in TNM8 is based on the presence of histological structures, and it has been shown that certain lymphatic features are used when assessing tumor nodules [13]. However, there are currently no guidelines as to what or how many specific features need to be present and whether some carry more weight than others, leaving this decision to the discretion of the pathologist and increasing the risk of high interobserver variation. As can be expected from a lack of guidelines, this study showed that histological features can guide pathologists towards a classification, but that they are not always sufficient. Nodules showing multiple features of a specific histological structure, such as a lymph node, vein, or nerve, were often classified as LNM, EMVI, or PNI. When nodules had no evidence of histological structures, pathologists mostly classified them as TD through a process of exclusion, which follows the definition provided by the TNM8. However, in nodules in which multiple histological structures were present, the final classifications varied considerably between pathologists. Furthermore, there were nodules with features of a single histological structure which pathologists would score as a TD instead of LNM, EMVI, or PNI. This is understandable as most histological features, such as lymphoid follicles or a fibrotic ring (i.e., a capsule), are non-specific. These difficult cases show that the definition of TD in the TNM8, which is based on the presence of many non-specific characteristics, is leaving room for subjectivity and interpretation.

Significant advances have been made in the classification of locoregional tumor spread since TNM5, with pathologists now attempting to apply proper scrutiny to tumor nodules and determine whether they are nodal or extranodal, rather than basing the distinction on arbitrary size or shape criteria which had no scientific basis. This increased scrutiny will inevitably lead to imperfect interobserver agreement since it is impossible to completely remove subjectivity from the professional opinion of each pathologist. However, this study has shown that pathologists are able to identify extranodal, and thus suspicious, tumor nodules with an overall agreement of 73.5%. Further subclassification into EMVI, PNI, and TD creates difficulties for many cases and thereby decreases interobserver agreement.

Increasing the complexity of a definition should be evidence-based, especially when it impairs interobserver agreement. Although there is evidence for the prognostic impact of different types of locoregional spread, this has only been analyzed for structures that are evidently recognized as, for example, EMVI or PNI. Also, the prognostic evidence for TD is based on meta-analyses that included TDs independent of their origin as these studies were performed prior to the implementation of the TNM8 [5]. For nodules that cannot easily be identified as LNM, EMVI, or PNI, and would therefore be called TDs, there is currently no evidence as to whether trying to classify them based on a possible origin has distinct effects on prognosis. Therefore, we suggest that all tumor nodules which are not easily recognizable as LNM, EMVI or PNI should be classified as TDs rather than separated into multiple categories. Since it has been suggested that TD could represent a stage of the invasion process [17–19], recording the presence of features suggesting a potential origin should be encouraged to help improve our understanding of tumor spread in the future, but should not determine the classification of the nodule at present.

The main limitation of this study is that pathologists were only able to review digital slides and could therefore not carry out additional techniques such as assessing deeper levels or elastin staining. Although elastin staining has shown to increase interobserver agreement with regard to venous invasion [20], it would have to be investigated whether the use of this technique would lead to a different final classification when classifying tumor nodules.

In conclusion, this study shows that the classification system for locoregional spread in rectal cancer has the potential to be improved. Refining the TNM definition of TDs further to build on the advances already made in the last decade would improve the prognostic homogeneity of patients grouped within one stage. For now, we suggest binary classification system for daily practice which classifies nodules as either nodal or extranodal which is more robust and evidence-based. This would improve risk stratification and avoid stage migration and inadequate treatment.

## Supplementary Information

Below is the link to the electronic supplementary material.

Supplementary file1 (DOCX 14 KB)
