# Supplementary material for: Interobserver variation in the classification of tumor deposits in rectal cancer—is the use of histopathological characteristics the way to go?
Source: Virchows Arch. 2021 Sep 4;479(6):1111–8. doi: 10.1007/s00428-021-03197-0 (PMC8724135; doi:10.1007/s00428-021-03197-0)
Supplement: Supplementary file 1 — Supplementary file1 (DOCX 14 KB) [file 428_2021_3197_MOESM1_ESM.docx]

**Supplementary table 1**

Interobserver agreement for different classifications and subgroups

|  |  |  |  |  |  | **Kappa agreement** | | |
| --- | --- | --- | --- | --- | --- | --- | --- | --- |
| **Patient group** |  | **Classification type** |  | **Mean percentage agreement** |  | 𝜿 |  | **95% Confidence Interval** |
| Overall (n=79) |  | LNM, TD, EMVI, PNI |  | 55.8% |  | 0.32 |  | (0.29-0.35) |
|  |  | Nodal vs Non-nodal |  | 76.1% |  | 0.40 |  | (0.36-0.44) |
|  |  |  |  |  |  |  |  |  |
| Without neoadjuvant treatment (n=50) |  | LNM, TD, EMVI, PNI |  | 52.2% |  | 0.27 |  | (0.23-0.31) |
|  |  | Nodal vs Non-nodal |  | 73.5% |  | 0.38 |  | (0.33-0.43) |
|  |  |  |  |  |  |  |  |  |
| With neoadjuvant treatment (n=29) |  | LNM, TD, EMVI, PNI |  | 61.9% |  | 0.40 |  | (0.35-0.44) |
|  |  | Nodal vs Non-nodal |  | 80.7% |  | 0.40 |  | (0.33-0.47) |

Agreement amongst pathologists when using a classification into 4 or 2 categories. Interobserver variation is shown for the whole cohort as well as the subgroups when cases were stratified to receiving neoadjuvant treatment.
